# A Novel Cognition-Guided Neurofeedback BCI Dataset on Nicotine Addiction

**DOI:** 10.3389/fnins.2021.647844

**Published:** 2021-07-06

**Authors:** Junjie Bu, Chang Liu, Huixing Gou, Hefan Gan, Yan Cheng, Mengyuan Liu, Rui Ni, Zhen Liang, Guanbao Cui, Ginger Qinghong Zeng, Xiaochu Zhang

**Affiliations:** ^1^Department of Radiology, The First Affiliated Hospital of USTC, Hefei National Laboratory for Physical Sciences at the Microscale and School of Life Science, Division of Life Science and Medicine, University of Science and Technology of China, Hefei, China; ^2^Department of Intelligent Medical Engineering, School of Biomedical Engineering, Anhui Medical University, Hefei, China; ^3^Department of Psychology, School of Humanities and Social Science, University of Science and Technology of China, Hefei, China; ^4^Department of Life Sciences, Imperial College London, London, United Kingdom; ^5^Institute of Advanced Technology, University of Science and Technology of China, Hefei, China; ^6^Hefei Medical Research Center on Alcohol Addiction, Anhui Mental Health Center, Hefei, China; ^7^Academy of Psychology and Behavior, Tianjin Normal University, Tianjin, China

**Keywords:** brain-computer interface, cognition-guided neurofeedback, nicotine addiction, electro encephalogram, public dataset

## Abstract

Compared with the traditional neurofeedback paradigm, the cognition-guided neurofeedback brain–computer interface (BCI) is a novel paradigm with significant effect on nicotine addiction. However, the cognition-guided neurofeedback BCI dataset is extremely lacking at present. This paper provides a BCI dataset based on a novel cognition-guided neurofeedback on nicotine addiction. Twenty-eight participants are recruited and involved in two visits of neurofeedback training. This cognition-guided neurofeedback includes two phases: an offline classifier construction and a real-time neurofeedback training. The original electroencephalogram (EEG) raw data of two phases are provided and evaluated in this paper. The event-related potential (ERP) amplitude and channel waveform suggest that our BCI dataset is of good quality and consistency. During neurofeedback training, the participants’ smoking cue reactivity patterns have a significant reduction. The mean accuracy of the multivariate pattern analysis (MVPA) classifier can reach approximately 70%. This novel cognition-guided neurofeedback BCI dataset can be used to develop comparisons with other neurofeedback systems and provide a reference for the development of other BCI algorithms and neurofeedback paradigms on addiction.

## Introduction

The brain–computer interface (BCI) is a hardware and software system integrated as the interface between the brain and the computer (Janapati et al., 2020). Considering time sensitivity and device portability, BCI system generally uses electroencephalogram (EEG), electrocorticogram (ECoG), functional magnetic resonance imaging (fMRI), functional near-infrared spectroscopy (fNIRS), magnetoencephalography (MEG), and positron emission tomography (PET) as imaging methods. Among them, EEG is the most widely used BCIs (Kwon et al., 2020). ECoG can collect purer signal than EEG, but it is invasive (Korostenskaja et al., 2014). Although fMRI and fNIRS have high spatial resolution, their temporal resolution is low (Cui et al., 2011). Besides, MEG and PET require large and expensive equipment and are not suitable for large-scale applications (Stam, 2010).

Nowadays, BCI system includes ERP, steady-state visually evoked potential (SSVEP), motor imagery (MI), and emotional BCI. In recent years, BCI-related fields have developed. Neurofeedback, as the predecessor of BCI, is applied to improve normal cognitive abilities, such as the enhancement of attention and working memory (Hsueh et al., 2016). It is also more and more used in the field of psychiatry studies, such as depression (Trambaiolli et al., 2021), anxiety (Gadea et al., 2020), addiction (Posson, 2019), etc.

Previous addiction-related neurofeedback datasets were usually based on fixed EEG or fMRI signals. Traditional EEG-based neurofeedback usually focuses on arousal/anxiety symptom in drug addiction by regulating α, α/θ, and sensorimotor rhythm (SMR)/β signals (Sokhadze et al., 2008). Real-time fMRI neurofeedback usually focuses on the activation of anterior cingulate cortex (ACC) or functional connectivity of bilateral ACC, medial prefrontal cortex (mPFC), orbitofrontal cortex (OFC), etc. (Martz et al., 2020). These datasets mentioned above have the following problems:

• No cognition task is performed to disclose the relationship between signals and behaviors/cognition components.
• Individual differences are not considered as all the subjects use a single and fixed signal to regulate the addiction behaviors.
• The efficacy of the traditional neurofeedback urges to be further improved in the treatment of addiction, as it is only rated as “probably efficacious.”

Therefore, it is of vital importance to propose a novel neurofeedback paradigm.

We attempt to resolve the shortcomings of traditional neurofeedback by proposing the EEG cognition-guided neurofeedback based on a cue reactivity model. According to this model, when smokers are presented with visual, taste, or tactile cues related to smoking, they will have significant cue reactivity (physiological and physical reaction). Similar reactivity is found in healthy control subjects to non-drug evocative stimuli (Garavan et al., 2000). Cue reactivity leads to impulsive behavior in drug-seeking behavior as well as relapse (Chiamulera, 2005). The cue reactivity task is usually used to induce cue reactivity of participants. In our study, we used a smoking cue reactivity task to induce specific cue reactivity. Cue reactivity has multiple EEG features including both time- (e.g., P300, slow positive wave) and frequency-domain (e.g., alpha oscillation) features. Compared with the single signal, combing with multiple features (both time and frequency domain) using multivariate pattern analysis can better enhance sensitivity of detecting a particular brain activity pattern (Littel et al., 2012; Campanella et al., 2014). Our cognition-guided neurofeedback regulated the multiple signals induced by the specific cue reactivity. Therefore, the cognition-guided neurofeedback process included a specific addiction-related cognitive model (cue reactivity model). Based on this model, we performed the cognitive task (cue reactivity task) to obtain the specific addiction-related brain activities represented by multiple EEG features. In addition, this paradigm achieved a good intervention effect for smokers: the number of cigarettes consumed per day decreased 30.6, 38.2, and 27.4% compared with the baseline (pre-neurofeedback) at 1 week, 1 month, and 4 months follow-up (Bu et al., 2019). Our BCI dataset is based on a novel neurofeedback paradigm, which is closed loop, individualized, and MVPA based. Previous researchers have proposed a framework for cognitive neurofeedback in food cravings (Sokunbi, 2014; Ihssen et al., 2017) and cocaine addiction (Kirschner et al., 2018), but most of these studies are based on fMRI.

According to the cue reactivity model, smoking behavior enhances the conditional value of smoking cues, which is specifically reflected at two levels (Supplementary Figure 1):

• Bottom–up automatic processing to activate attentional functions.
• Top–down modulation of sensory inputting and motor controlling from cortical area.

According to conditioned reflex learning theory (Rees and Heather, 1995; Versace et al., 2017), the craving for cigarettes induced by smoking cues may partly relate to the conditioned reflex established by learning to associate smoking-related cues with smoking behavior (Karelitz, 2020). The reinforcement of smoking behavior also enhances the conditioned value of smoking cues, which automatically activates the attention function from the bottom to up. Besides, previous fMRI study has reported the regulation of cue-induced cravings from the top to down (Li et al., 2013).

Different from other neutral neurofeedback (for example, the height of the column is used for neurofeedback visual signals) (Zotev et al., 2014), we used an adaptive closed-loop method to develop our cognition-guided neurofeedback in the process of self-regulation training (Bu et al., 2019). The essence of this method is that when the participants’ task performance decreased, the program would arouse the participants’ attention and alertness by increasing the difficulty of the current task (deBettencourt et al., 2015). We established a mapping relationship between the probabilistic score and the 11 pictures (the probabilistic score was positively correlated with the desiring rating of the picture). Participants’ brain activity state toward smoking cues was reflected as smoking-related pictures with different craving levels. At the same time, the picture would affect the brain activity pattern in the next trial, which was mapped to the corresponding smoking-related picture. In other words, we amplified the consequence of neurofeedback training: rewarding successful downregulation by reducing difficulty and punishing unsuccessful downregulation by increasing difficulty to activate participants’ self-monitoring ability. By this way, an adaptable closed-loop effect was formed, which is one of the characteristics of our cognition-guided neurofeedback.

Neurofeedback intervention methods may produce different effects in different participants. Different visits of the same participant will also lead to different EEG signals (Gruzelier, 2014). Considering the individual differences of neurofeedback intervention and changeable craving of a specific participant, our neurofeedback paradigm built an individual model for each visit of each participant, which eliminated individual differences to a great extent. Each subject had exclusive classifier, and the regulated EEG signals (time and frequency domain) were obtained from his/her own cue reactivity task. The model used by the subject was reconstructed in the current cycle. Traditional neurofeedback regulated fixed signal, which may be not suitable in some subjects. Individual classification is one of the characteristics of our cognition-guided neurofeedback.

Traditional dependent neurofeedback usually includes single signal. There are also researches about neurofeedback based on network features, such as the algorithm based on common spatial pattern (CSP) and local characteristic-scale decomposition (LCD) developed by Ai et al. (2019). Addiction is a complex pattern of brain activity, rather than just being related to a certain electrical brain signal, such as P300 (Littel et al., 2012) and α power (Cui et al., 2013). The development of a classifier with more features or variables is particularly important. MVPA classifier is used to extract features of different dimensions from cue reactivity task (Zafar et al., 2017). This algorithm based on machine learning can detect the brain reactivity patterns in response to smoking and neutral stimulus more sensitively (Sitaram et al., 2017).

Our novel paradigm has a good performance and shows significant short- and long-term effects (Bu et al., 2019), but so far, there is a lack of novel neurofeedback-related datasets. Sharing these datasets online can facilitate comparison with other neurofeedback datasets, promote parameter optimization process, and help optimize the BCI algorithm. Single data may be limited. More datasets shared online can also be applied to big data models to get better results. In addition, BCI datasets are also instructive for BCI hardware development. In the field of psychiatry, more datasets on clinical nicotine addiction are needed to promote the study of addiction mechanisms, especially smoking cue reactivity mechanisms.

In this paper, we provide a novel dataset based on a cognition-guided, closed-loop, and individualized neurofeedback, which is based on MVPA classifier. The dataset includes EEG data of two phases: the cue reactivity task and the real-time neurofeedback training. In addition, we evaluated the quality of EEG data in our dataset by ERP and topographic map analysis. A linear correlation is used to indicate the trend of probabilistic score in neurofeedback training. Prediction accuracies of each participant are used to evaluate the classification power. The publication of novel cognition-guided neurofeedback dataset is of vital importance to the development of this field.

## Materials and Methods

### Participants

In this study, 28 participants (male; mean age, 23.7 years) were recruited through online advertisements and posters by the criteria listed below. The score of Fagerstrom Test for Nicotine Dependence (FTND) of the 28 subjects were 4.6 ± 1.9 (mean ± SD, Table 1). Since there are only a few female smokers (2.7%) in China, we recruited only male participants for this experiment. This study was approved by the Human Ethics Committee of the University of Science and Technology of China (USTC). According our records, no participant reported uncomfortable feelings after neurofeedback training (Bu et al., 2019).

**TABLE 1:** Demographic information of 28 participants.

**Characteristic**	**Value**
Age (years)	23.7 (3.8)
Education (years)	14.8 (2.5)
Cigarettes (day)	14.1 (4.5)
Cigarette use (years)	7.1 (3.9)
FTND score	4.6 (1.9)

**Values were mean and values in parentheses were 1 standard deviation (SD)*.*

Selection criteria were as follows:

• Smoking 10 cigarettes or more per day for at least 2 years
• Right-handedness
• 18–40 years old
• Normal or corrected-to-normal vision
• Normal mental and physical health condition assessed by the Mini-International Neuropsychiatric Interview (MINI).

Exclusion criteria were as follows:

• Chronic neurological, psychiatric, or medical disease
• Taking any drugs in the past 3 months
• Unable to perform EEG for any reasons.

### Experimental Design

**FIGURE 1:**
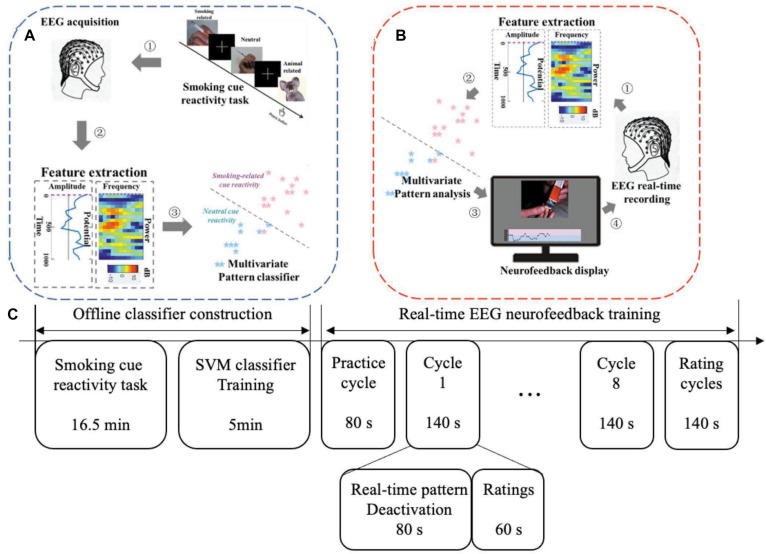
Two phases for one neurofeedback visit: offline model construction and real-time neurofeedback training. **(A)** Offline classifier construction phase. **(B)** Real-time EEG neurofeedback training phase. **(C)** Experimental protocol in one visit. NF, neurofeedback.

We developed a novel cognition-guided neurofeedback paradigm on nicotine addiction. Participants were involved in two continuous visits over 2 days. In each visit, participants experienced a two-phase procedure shown in Figure 1C: offline classifier construction and real-time EEG neurofeedback training.

**FIGURE 2:**
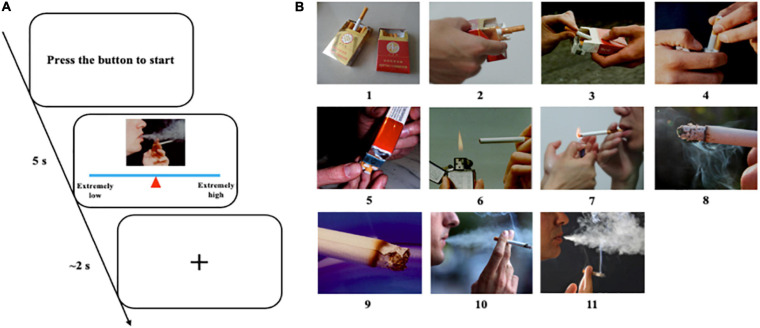
Evaluation of nicotine related cues. **(A)** Picture evaluation protocol and procedure. Participants pressed the button to start a new trial. They had 5 s to move the mouse to change the position of the triangle on the line to give an appropriate score according to their craving to this picture. Then, they had about 2 s to rest and wait for the next trial. **(B)** Eleven selected pictures listed in ascending order of craving score.

Before the formal experiment, we prepared 330 pictures (210 × 180 pixels), including 150 smoking-related pictures (e.g., holding a cigarette in hand), 150 paired neutral pictures (e.g., holding a pencil in hand), and 30 pictures of animals (e.g., cat). The selection of these pictures mainly referred to the previous work on addiction of our laboratory (Zhang, 2011). To eliminate the possible impact of visual information, these smoking pictures and neutral pictures were matched as much as possible in terms of visual information such as background color, brightness, and object orientation. We recruited 20 participants to evaluate the craving degree of these pictures. The picture evaluation procedure is shown in Figure 2A. Eleven pictures were selected for real-time EEG neurofeedback training from 150 smoking-related pictures. They were listed in ascending order of the craving score given by the participants (Figure 2B).

#### Offline Classifier Construction

Offline classifier construction consisted of two parts: the smoking cue reactivity task and the support vector machines (SVMs) classifier training (Figure 1A). EEG data collected from cue reactivity task were used to train the classifier.

The smoking cue reactivity task was block design. Three smoking and neutral blocks each were assessed in a pseudo-randomized order (neutral, smoking, smoking, neutral, smoking, and neutral). There were 55 trials in each block, and participants were requested to focus on the pictures as much as possible. Among them, 50 trials were smoking or neutral cues. Another five randomly distributed trials were animal cues, which were used to improve the concentration of participants on the task. They were asked to press the space button of the keyboard quickly and accurately when seeing animal cues (the main objects are animals, such as cats and dogs) and press the space button of the keyboard quickly and accurately. At the same time, EEG data were collected during each block. In the block interval, participants had 90 s of rest. The smoking cue reactivity program was written by Psychophysics Toolbox^1^ for MATLAB (version 2016a, MathWorks Inc., Natick, MA, United States).

EEG data were processed offline in order to remove noise using EEGLAB (more details and parameters will be mentioned in *Data processing*). Permutation test was performed to extract features in time and frequency domain from the processed data (α = 0.05). Specifically, we compared EEG data collected from smoking and neutral blocks to obtain significantly different features in time (amplitude) and frequency (power) domain. These statistically significant features were transferred to cluster features via cluster-based statistic (maximum cluster-level mass) and imported into the classifier in the form of a column vector to train a personalized SVM classifier to recognize brain activity patterns in response to smoking stimuli. This step was calculated using the MATLAB function fitcsvm (Bu et al., 2019). Sixty channels (64 standard channels except for CB1, CB2, HEOG, and VEOG) were used to construct the model. In order to evaluate the classification effectiveness of the classifier, we used 20% of the trials to calculate the prediction accuracy in each cross-validation cycle. This step repeated five times for each participant.

#### Real-Time EEG Neurofeedback Training

**FIGURE 3:**
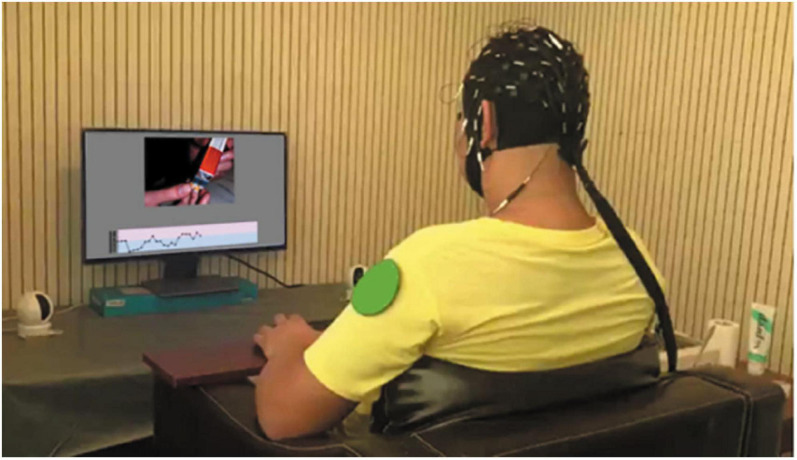
One scene of a participant in the phase of neurofeedback regulation training. A participant is wearing EEG cap and watching neurofeedback display. A camera to monitor the status of the participant.

The neurofeedback training phase comprised eight same cycles in total, including 40 trials (80 s) and a rating (60 s) in each phase. Before the first trial, we carried out a practice cycle to make sure that participants understand the experimental requirements (Figure 3).

The real-time neurofeedback signal of each trial was updated every 2 s, including 1 s of real-time EEG acquisition and classification each. Online preprocessing (the same with offline processing mentioned above) was performed to remove noise. Processed EEG raw data were put into the classifier. Based on the SVM classifier, a probabilistic score (range, 0–1) was returned to characterize the similarity probability between the current brain activity and the brain activity pattern of smoking measured in smoking cue reactivity task. The probabilistic score was presented to participants in real time as a moving point in the feedback line at the bottom of the screen (Figure 1B). In order to prevent drastic changes of the line, the value of each point was obtained by averaging the value of the current point and the previous 2 points, and the first 2 points in the line were fixed 0.5 (no previous points to be averaged). At the same time, based on this value, the corresponding type of picture was displayed on the top of the screen according to adaptive closed-loop design. A higher probabilistic score corresponded to a picture with a higher craving level. This closed-loop neurofeedback program was written by Psychophysics Toolbox for MATLAB.

In this phase, participants were asked to downregulate the line repeatedly and continuously while fixating the picture. If one strategy failed to downgrade the line well, they needed to change the strategy until they found the most effective one. After finishing training and rating cycles (Figure 1C), participants were asked to record 10 mental strategies that may effectively downregulate the neurofeedback signals. To improve the initiative of participants, we informed them about the rewards they can get for completing the experiment in advance. Participants who completed eight cycles would receive a reward of up to 140 RMB according to their performance (proportional to mean probabilistic score). After completing a cycle, participants would have a rest for 1 min.

### Data Acquisition

EEG raw data were collected using a SynAmps RT amplifier (NeuroScan, Inc., Sterling, VA, United States), and an electrode cap was with 64 Ag/AgCl electrodes located according to international 10–20 system. Additionally, the left (M1) and right mastoids (M2) were also recorded. The impedance of all electrodes was kept under 5 kΩ based on the reference electrode attached to the tip of the nose. The EOG was recorded by VEOG (above and below the left eye) and HEOG (lateral to the outer canthi of both eyes) using four electrodes (VEOL, VEOU, HEOL, and HEOR). In order to reduce the influence of electromagnetic interference on EEG signals, we grounded the AFz electrode. The sampling rate of the EEG raw data was 250 Hz. In neurofeedback sessions, EEG raw signals were collected with the same parameters and sent to MATLAB program using NeuroScan Access SDK (NeuroScan, Inc., Sterling, VA, United States).

### Data Processing

The EEG raw data processing was conducted by EEGLAB toolbox (version 14_1_1b) for MATLAB. The preprocessing steps included high-pass filter (0.5 Hz), epoch (−200 to 1,000 ms, −200 to 0 ms as prestimulus interval to conduct baseline correction), and blink artifacts (using a conventional recursive least squares algorithm). ERP analysis was also conducted in EEGLAB. Epochs containing the amplitude changes exceeding ±100 mV were rejected. The ERPs were grand averaged based on different types of stimulus (smoking and neutral) across participants. The power characteristics of the time–frequency domain were mainly obtained based on the wavelet analysis algorithm. The frequency of the EEG data was divided into five ranges: alpha, low beta, high beta, low gamma, and high gamma waves.

### Data Evaluation

At the offline classifier construction phase of the cue reactivity task, EEG raw data were collected, and the signal-to-noise ratio (SNR) of Pz channel was calculated to verify the quality of the data (Hu et al., 2010). EEG epochs from −200 to 1,000 ms were preprocessed and averaged to calculate ERP waveforms separately for two visits. Besides, topographic maps were shown every 200 ms in the time window of 0–1,000 ms. Processed EEG data were imported to the classifier, and fivefold cross-validation accuracy was used to calculate its prediction accuracy.

In neurofeedback training phase, 1-s EEG data were preprocessed and shown at only channels located on the frontal midline. Topographic maps were shown every 200 ms in the time window of 0–1,000 ms. These 1-s EEG data were inputted to the personalized classifier model built in the offline phase, and a probabilistic score of smoking cue reactivity patterns was given. The score reflected the matching degree between the participant’s current brain activity pattern and the pattern while viewing smoking cues in cue reactivity task. A high score indicated closely matched patterns. After training, the correlation between decreased P300 amplitudes (pre–post) and decreased craving score [score of tobacco craving questionnaire (TCQ), pre vs. post] was performed.

### Statistical Analysis

Statistical analysis was conducted in Statistics and machine learning toolbox in MATLAB. The comparison of features was performed with a non-parametric permutation test. The correlation of probabilistic score and training cycles was analyzed using Pearson’s correlation. The comparison between the prediction accuracy and chance level used one-sample Student’s *t*-test. The normality test was based on Kolmogorov–Smirnov (KS) test. Fivefold cross-validation was used to calculate the classification accuracy of the classifier. The reported *p*-values were all two-tailed. The significant threshold α is 0.05.

## Results

### EEG Data Evaluation

The database is 3.96 GB, including 2.44 GB EEG data of cue reactivity task, 1.52 GB EEG data of neurofeedback, 16.8 kB demographic data, and 1.81 kB channel location file. Twenty-eight subjects were included in this database. The cue reactivity data were divided into two files: “cue reactivity_1.zip” and “cue reactivity_2.zip.” Each subject had two runs, and each run contained six CNT files, which could be read in EEGLAB toolbox. The neurofeedback data were in “nf_eeg.zip.” Each subject had two runs, and each run contained eight EEG MAT files (channel^∗^time), which can be read in MATLAB. To facilitate subsequent data processing, electrode-position file and marker information were also provided. The channel location file named “chan62.zip,” the baseline demographic and clinical characteristics named “baseline.mat,” and trigger information file named “trigger.md” can be found in the root directory.

**FIGURE 4:**
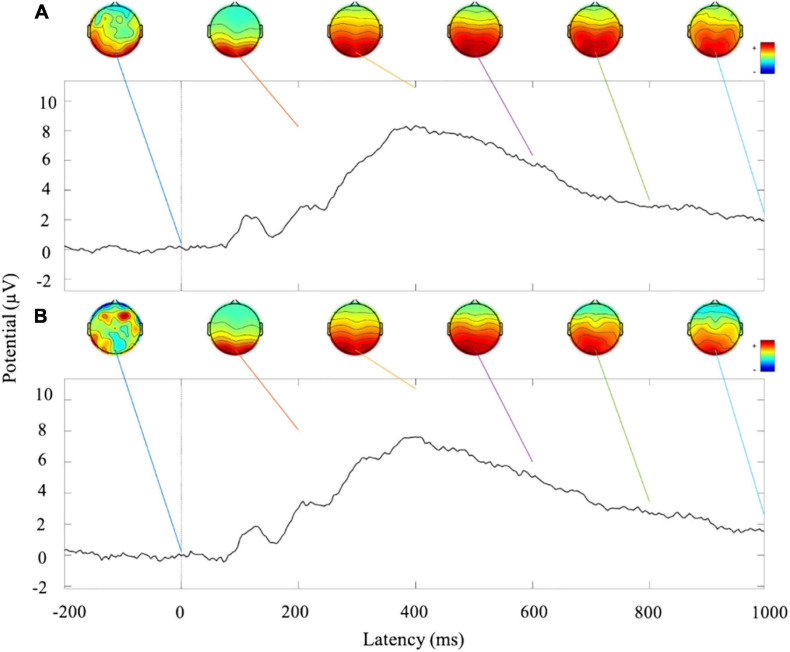
Analysis of ERP amplitude and topographic map of cue reactivity task. **(A)** ERP waveform and topographic maps of the first visit of cue reactivity task phase. **(B)** ERP waveform and topographic maps of the second visit of cue reactivity task phase.

**FIGURE 5:**
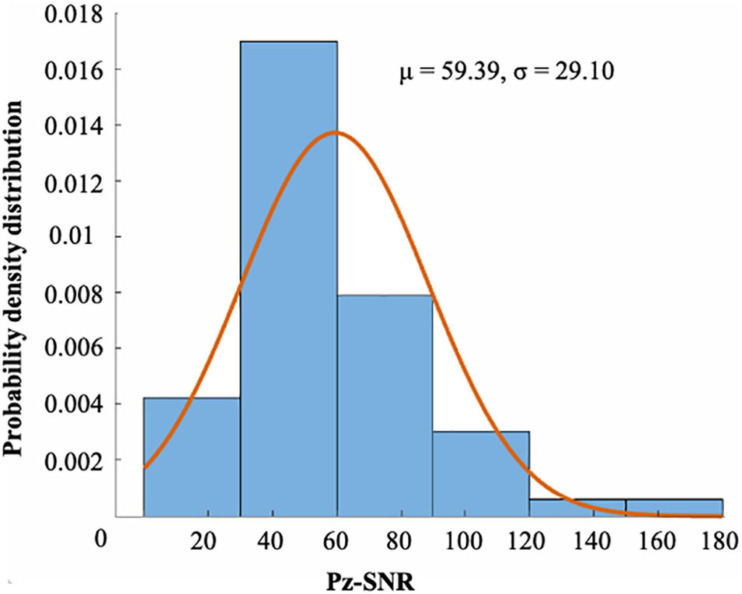
The distribution of the SNR of Pz channel (μ = 59.39, σ = 29.10). There were 55 runs of 28 subjects. There was no significant difference between the distribution and the normal distribution by KS test (*p* = 0.29).

Figures 4A,B show the ERP amplitude and topographic map of cue reactivity task in the first and second visits. The data of the two visits have good consistency. The P300 component of ERP caused by addictive substance-related cues is usually a feature of substance use disorders, and the P300 amplitude is found to be related to the craving for smoking (Littel et al., 2012; Campanella et al., 2014). In our research, the P300 ERP component induced by smoking cues can be observed at approximately 300–550 ms. We evaluated the distribution of the Pz-SNR (Figure 5). KS test was performed to check the normality of distribution. There was no significant difference between the data distribution and the normal distribution (*p* = 0.29).

**FIGURE 6:**
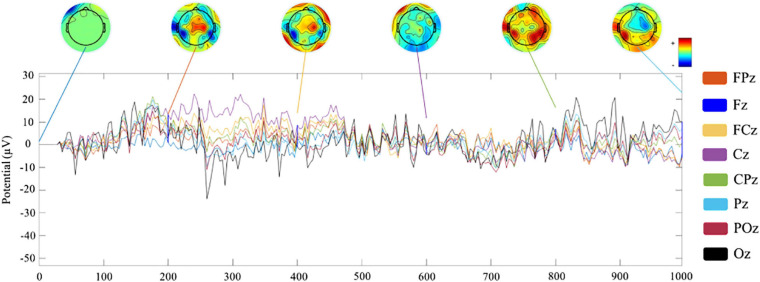
Channel waveform and topographic map of real-time neurofeedback training phase.

Channel waveform and topographic map of preprocessed 1-s real-time neurofeedback training data of eight channels (including FPz, Fz, FCz, Cz, CPz, Pz, POz, and Oz) are shown in different color to express brain electrical activity. We used the data of the 10th trial, the first cycle, the first visit (run1) of subject 1 (s1) as an example (Figure 6).

### Feature Extraction Evaluation

**FIGURE 7:**
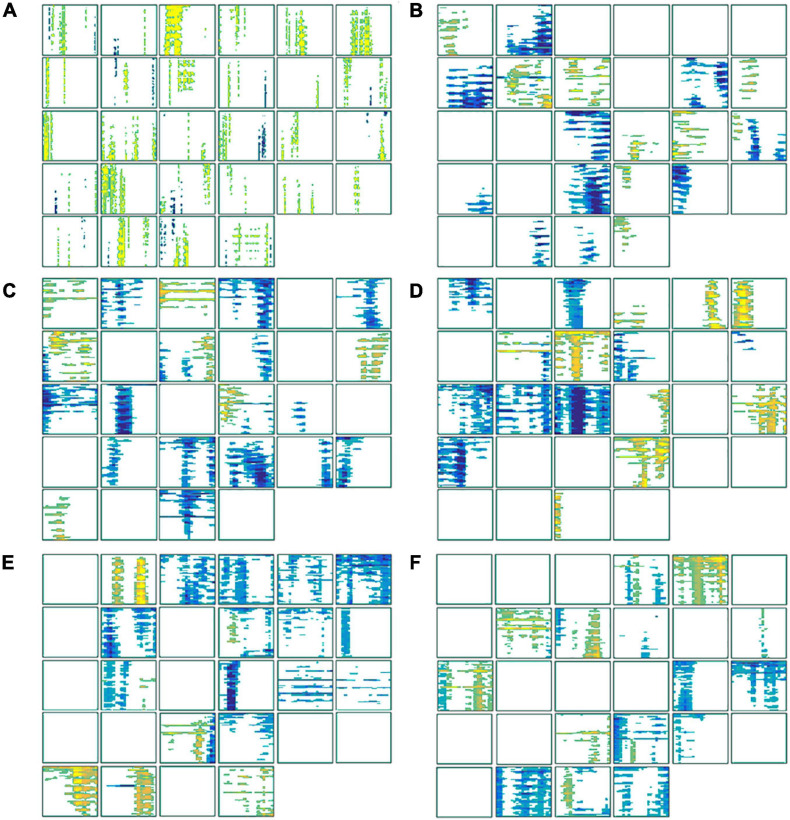
The statistically significant features used to construct the classifier. **(A)** The features of each subject in the time domain; **(B)** the features in the alpha (8–13 Hz) frequency band; **(C)** the features in the low beta (14–20 Hz) frequency band; **(D)** the features in the high beta (21–30 Hz) frequency band; **(E)** the features in the low gamma (31–48 Hz) frequency band; **(F)** the features in the high-gamma (52–80 Hz) frequency band.

These time (Figure 7A) and frequency domain (Figures 7B–F) features from 28 participants were used to construct the classifier. For each block diagram, the abscissa represents the time information (ms), and the ordinate represents electrode channel information (60 channels) respectively. The yellow and blue areas in the figure represent the characteristic signals that were statistically significant with smoking > non-smoking conditions and the characteristic signal that was statistically significant with smoking < non-smoking conditions (α = 0.05), respectively.

### Neurofeedback Performance Evaluation

**FIGURE 8:**
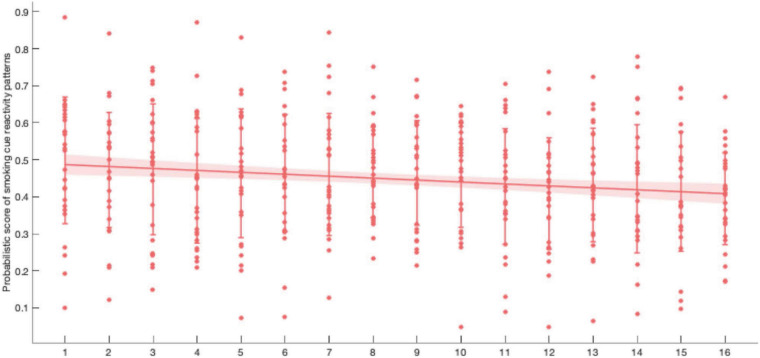
Within two visit of neurofeedback learning, participants tried to reduce the probabilistic score (*r* = −0.1545, *p* = 0.0010). Error bar: SD; shaded area: 95% CI.

Participants were trained for 16 cycles in two visits. In each trial of the neurofeedback cycle, the average score of 40 probabilistic scores was recorded as neurofeedback performance in the respective cycle. We performed a linear regression analysis on the probabilistic score and the training cycles. A significant negative correlation (*r* = −0.1545, *p* = 0.0010) was found, which showed that the match probability (the current brain activity patterns and the patterns when viewing smoking cues) decreased with training progressed (Figure 8). In other words, the difference in brain activity patterns between viewing the smoking cues and the neutral cue was smaller after neurofeedback training.

### Classifier Power Evaluation

**FIGURE 9:**
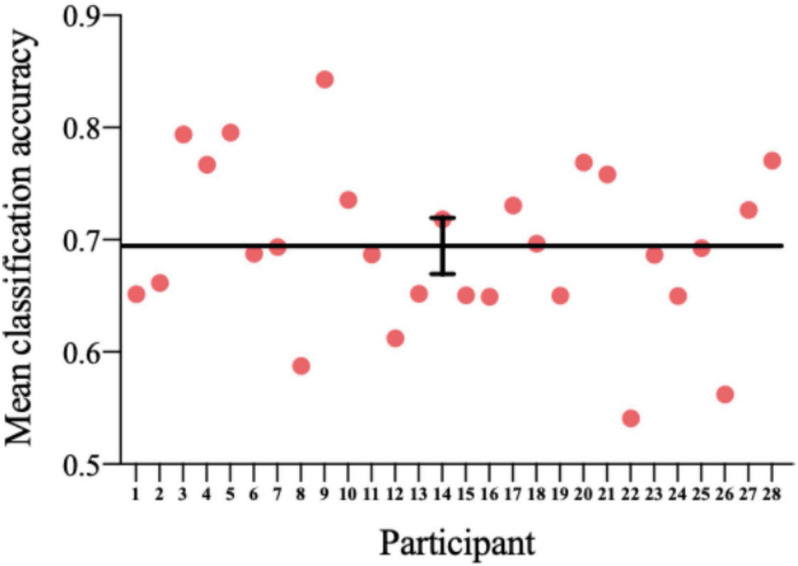
The accuracy of the classifier for each participant. Horizontal line: average; error bar: SE.

Each point in Figure 9 represents fivefold cross-validation accuracy of one participant. The accuracy of the classifier varies between 0.5409 and 0.8427, with a mean accuracy of 0.6935. One-sample *t*-test showed a significant difference between the prediction accuracy and the chance level (*p* = 4.4176 × 10^–14^).

## Conclusion and Discussion

In this paper, we developed a novel cognition-guided neurofeedback paradigm. Neurofeedback technology is an effective method to regulate brain signals and neuroplasticity, which involves brain networks of reward, control, and learning (Sitaram et al., 2017). Among them, dorsolateral prefrontal cortex (dlPFC) and posterior parietal cortex (PPC) in the control network will be activated during the execution of the strategy, and the learning network [mainly includes the dorsal striatum (DS)] is responsible for strategy learning in neurofeedback. The theoretical models of neurofeedback learning include operant (or instrumental) learning, motor learning, dual process theory, awareness theory, global workspace theory, and skill learning theory (Sitaram et al., 2017). These theoretical models are not mutually exclusive but compatible. Operant learning can be regarded as part of the dual process theory, which includes automatically process and controlled process (Enriquez-Geppert et al., 2017). Normally, approximately 15–30% of subjects do not respond to neurofeedback/BCI (Weber et al., 2011). Strategies are extremely important for neurofeedback. In the process of neurofeedback learning, strategies may promote or hinder neurofeedback learning, depending on the appropriate degree of scheduling cognitive resources in the process (Gaume et al., 2016; Davelaar, 2018).

**FIGURE 10:**
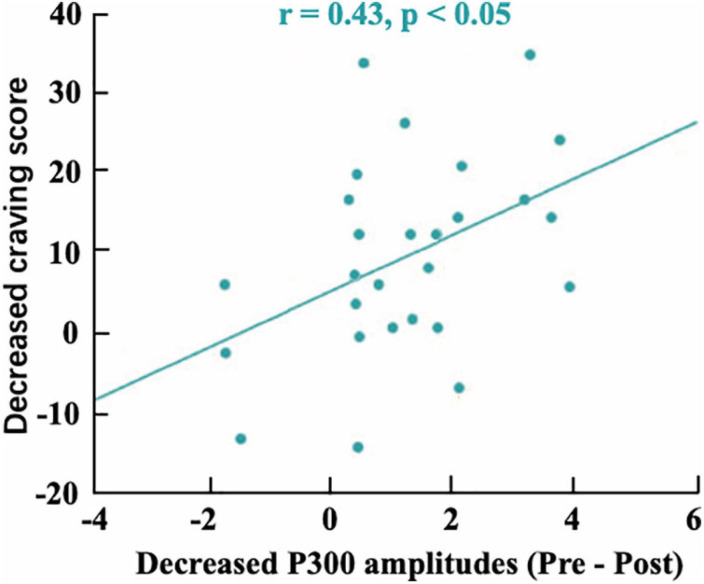
The correlation between the mean decreased P300 amplitudes and decreased craving score (*r* = 0.43).

In our implicit neurofeedback, the significant negative correlation (*r* = −0.1545, *p* = 0.0010) of the probabilistic score and training cycles demonstrates good neurofeedback learning effect. It also shows that the brain activity pattern in response to smoking stimuli represented by EEG signals can be successfully downregulated after two neurofeedback trainings. This is similar to previous rt-fMRI neurofeedback studies based on visual perception and attention brain activity patterns (deBettencourt et al., 2015; Amano et al., 2016). Our research also shows that the changes in brain patterns influenced addictive behaviors. The subjects’ TCQ craving score and P300 amplitude decreased significantly after neurofeedback training (Bu et al., 2019), and there was a positive correlation between them (Figure 10). The results revealed the relationship between neural signals and behavioral indicators.

In particular, previous neurofeedback studies based on the regulation of brain activity patterns focused on training normal subjects to improve their cognitive abilities. The results of our study further extended these findings to patients with mental illness (smoking addicts), which also had the potential to regulate brain activity patterns. Our dataset provided important support for the extension of neurofeedback training of multivariable brain activity pattern regulation to clinical research in the future.

This dataset is of high quality and good integrity. In order to reduce artifacts as much as possible and improve the SNR, we added simple cognitive activities while performing the cue reactivity task to ensure that the collected data were brain electrical signals from the experiment rather than distractions. Additionally, in the cue reactivity task phase and the neurofeedback training phase, an intertrial interval (ITI) was used to eliminate the detention effect of the previous trial and gave participants a certain time to rest until they felt comfortable enough for the next trial. During the experiment, participants were instructed to blink and swallow as little as possible to obtain purer EEG raw data. As can be seen in Figure 4, ERP waveforms and topographic maps have strong consistency and repeatability between visits. In addition, we checked the strategies reported by the participants and the surveillance video during neurofeedback and found that no subjects used the strategies that were not allowed, such as not watching the screen. The decline in smoking cue reactivity patterns and the relatively high model prediction accuracy provided a guarantee for the intervention effect and method reliability of neurofeedback BCI. Nevertheless, the dataset also had some limitations. Considering that the prevalence among female smokers in China was very low (2.7%), we only recruited male participants. The dataset we present is the first cognition-guided neurofeedback BCI dataset on nicotine addiction to our knowledge. Participants completing two separate visits of training showed improvement in smoking cue reactivity patterns. High-quality EEG raw data are provided, and the classifier is evaluated and proven to have relatively high classification accuracy. Our two-visit, cognition-guided neurofeedback BCI can be compared with other neurofeedback paradigms to develop new neurofeedback systems.

Our previous research extracted the characteristics of the EEG signal in the time and frequency domains for SVM modeling. As far as we know, the cue reactivity task is commonly studied in substance addiction (Hardy et al., 2017). The dataset we provided contains the participants’ brain electrical activity exposed to both smoking-related cues and neutral cues. There are other methods to analyze the dataset, such as the microstate (Michel, 2018) and source analysis (Zhou et al., 2019). These methods can be implemented on this dataset to discover mechanisms of nicotine addiction. The EEG data provided in this study can be used to verify other existing models or optimize parameters. In addition, the dataset can be used to development other ERP-based BCI algorithms.

In future research, BCI datasets can still be improved from the following aspects. First, a higher number of channels and sampling rate can be applied to obtain higher BCI bandwidth. Second, the extraction of feature can be improved. Different network connection of participants toward smoking and neutral cues can be incorporated into classification features to obtain a higher degree of discrimination (Ai et al., 2019). Third, SVM classifier was used in this study, and a relatively high classification accuracy rate was obtained. Subsequent studies can use other machine learning method to obtain higher model prediction rates, such as logistic regression and decision tree. For example, a feature extraction method was developed based on autoregression and used random forest classifiers to identify the EEG features of patients with epilepsy with a best accuracy of 97.352% (Zhang et al., 2017). Fourth, this study conducted two visits of neurofeedback training, which was mainly based on skill learning theory (Hinterberger et al., 2005; Koralek et al., 2012) and previous MRI research (Young et al., 2017a,b). In future BCI studies, longer training periods can be used to evaluate the impact of training time on neurofeedback effects. Finally, this study used a simple floating line as the visual form of neurofeedback. Nowadays, BCI is moving in the direction of gamification (de Castro-Cros et al., 2020), which is also the direction for future BCI datasets.

In conclusion, our novel neurofeedback BCI dataset has a significant contribution to this field. We offer the community access to our EEG data from our BCI experiment. The neurofeedback protocol that we developed and applied is based on the long-term research on nicotine addiction of our group and will act as a reference for subsequent neurofeedback BCI research.

## Data Availability Statement

The datasets presented in this study can be found in online repositories. The names of the repository/repositories and accession number(s) can be found below: https://github.com/Qingqingran/Cognition-guided-neurofeedback-BCI-dataset-on-nicotine-addiction/releases/tag/publish.

## Ethics Statement

The studies involving human participants were reviewed and approved by the Human Ethics Committee of the University of Science and Technology of China. The patients/participants provided their written informed consent to participate in this study. Written informed consent was obtained from the individual(s) for the publication of any potentially identifiable images or data included in this article.

## Author Contributions

JB and XZ contributed to the experimental design. JB and GC contributed to the data collection. JB, CL, HGo, HGa, YC, ML, GQZ, and XZ contributed to the manuscript writing. CL, RN, and ZL contributed to the data processing. CL and HGo contributed to the data uploading. All listed authors made direct contributions to the article and approved it for publication.

## Conflict of Interest

The authors declare that the research was conducted in the absence of any commercial or financial relationships that could be construed as a potential conflict of interest.

## Footnotes

1 http://psychtoolbox.org/
