# Supplementary material for: A Novel Cognition-Guided Neurofeedback BCI Dataset on Nicotine Addiction
Source: Front Neurosci. 2021 Jul 6;15:647844. doi: 10.3389/fnins.2021.647844 (PMC8290081; doi:10.3389/fnins.2021.647844)
Supplement: Supplementary file 1 [file Data_Sheet_1.docx]

Supplementary Material

# Cue reactivity model

Cue reactivity model reveals main neural pathways related to the smoker's cue reactivity (Chiamulera, 2005). Bottom-up emotional and motivational stimulus are processed through amygdala and hippocampus to the mesolimbic pathway so as to induce physiological and physical reaction. Top-down process integrates emotion, motivation, attention processing and cognitive information so as to realize attention shift, execute and control reaction output.


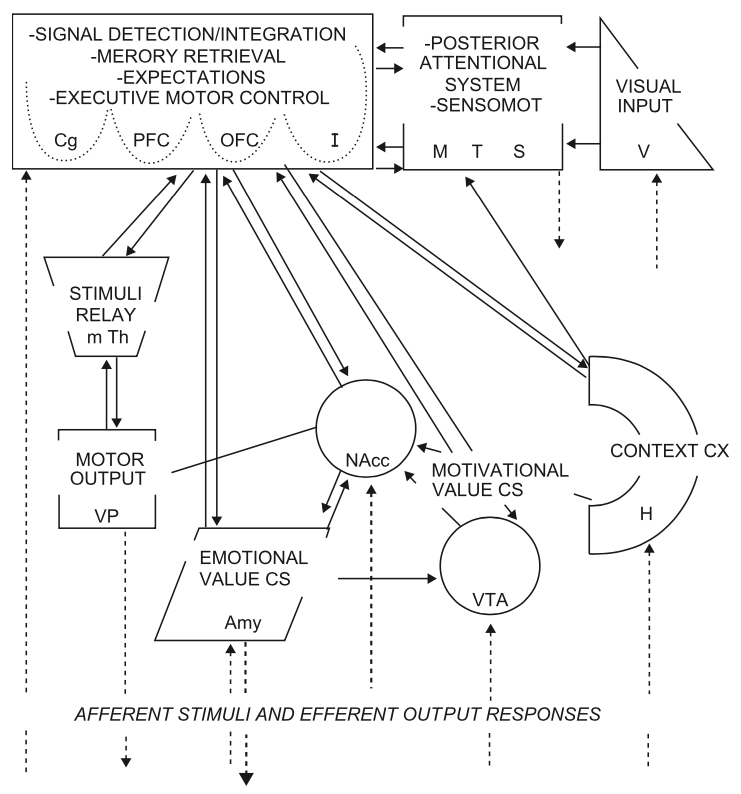


**Supplementary Figure 1.** A schematic diagram that reveals the main neural pathways and functions in the cue reactivity of smoking (Chiamulera, 2005). Abbreviations: CG, cingulate; PFC, prefrontal cortex; OFC, orbitofrontal cortex; I, insula; M and S, sensorimotor cortex; T, temporal cortex; V, visual cortex; mTh, medial thalamic nuclei; VP, ventrostriatal regions; NAcc, nucleus accumbens; H, hippocampus; Amy, amygdala; VTA, ventral tegmental area; CS, conditioned stimuli; CX, contextual stimuli; SENSOMOT, sensorimotor functions.

Reference:

Chiamulera, C. (2005). Cue reactivity in nicotine and tobacco dependence: a “multiple-action” model of nicotine as a primary reinforcement and as an enhancer of the effects of smoking-associated stimuli. *Brain Res. Rev.* 48, 74–97. doi:10.1016/j.brainresrev.2004.08.005.
